# Seizure-Associated Takotsubo Cardiomyopathy

**DOI:** 10.7759/cureus.10599

**Published:** 2020-09-22

**Authors:** Juliette Conte, Michael J Yoo, Neil P Larson

**Affiliations:** 1 Emergency Medicine, Brooke Army Medical Center, Fort Sam Houston, San Antonio, USA

**Keywords:** takotsubo, cardiomyopathy, seizure, systolic heart failure

## Abstract

Takotsubo cardiomyopathy (TCOM) is a syndrome characterized by acute systolic dysfunction that can mimic acute coronary syndrome (ACS), usually incited by physical or emotional stress. However, acute neurological dysfunction, including seizures, has been recently described as an additional risk factor for the development of TCOM. This specific case report reviews the pathophysiology of TCOM and its management. We emphasize that providers should maintain a high index of suspicion for TCOM after acute neurologic dysfunction in patients with chest pain or hemodynamic instability, while also initiating proper investigation for ACS. Although classically thought of as a transient process, recent data show that both in-hospital and post-hospital morbidity and mortality related to this condition remain concerning.

## Introduction

Takotsubo cardiomyopathy (TCOM) has been historically associated with the development of transient, acute heart failure following physical or emotional distress. TCOM is characterized by acute systolic dysfunction that does not correlate with a regional coronary artery perfusion territory, occurring in the absence of angiographic evidence of obstructive coronary artery disease (CAD). As research continues, the list of known inciting risk factors for TCOM has expanded and includes acute neurologic dysfunction [1,2]. While the clinical heart failure syndrome caused by these differing inciting factors currently remains a single entity, prognosis and outcomes vary by underlying inciting event, with acute neurologic dysfunction demonstrating worse long-term outcomes [2]. We present a case of a 48-year-old woman with a past medical history of epilepsy who developed TCOM after a breakthrough seizure, with the onset of symptoms occurring during her ED course. The authors highlight that development of anginal, anginal equivalent, or acute heart failure symptoms after a seizure should raise suspicion for TCOM and warrants a full cardiac workup. Specifically, for patients meeting ACS criteria, even in the setting of acute neurologic dysfunction suggesting TCOM, cardiac catheterization should be performed to rule out myocardial infarction or acute plaque rupture.

## Case presentation

A 48-year-old woman with a past medical history of epilepsy presented to the ED in a postictal state after a witnessed seizure. The patient’s husband reported a history of encephalomalacia from prior arteriovenous malformations and coagulopathy secondary to a prothrombin gene mutation. He described that the seizure activity had lasted less than a minute, with a generalized tonic-clonic pattern, consistent with her last breakthrough event seven years prior. A review of systems obtained after the patient became more lucid was negative for medication non-compliance, recent infections, illicit drug use, trauma, or headache but was positive for consumption of one alcoholic beverage three hours prior to her seizure.

On arrival, the patient’s vital signs included blood pressure of 157/100 mmHg, heart rate of 136 beats per minute, 20 respirations per minute, oxygen saturation of 97% on room air, and temperature of 97.3 °F. Her physical examination was unremarkable with a non-localizing neurological examination. The initial workup including a non-contrasted CT of the head was negative for acute findings. Laboratory evaluations including a complete blood cell count, metabolic panel, and thyroid function tests were only notable for an elevated leukocyte count of 15,700 cells/microliter.

**Figure 1:**
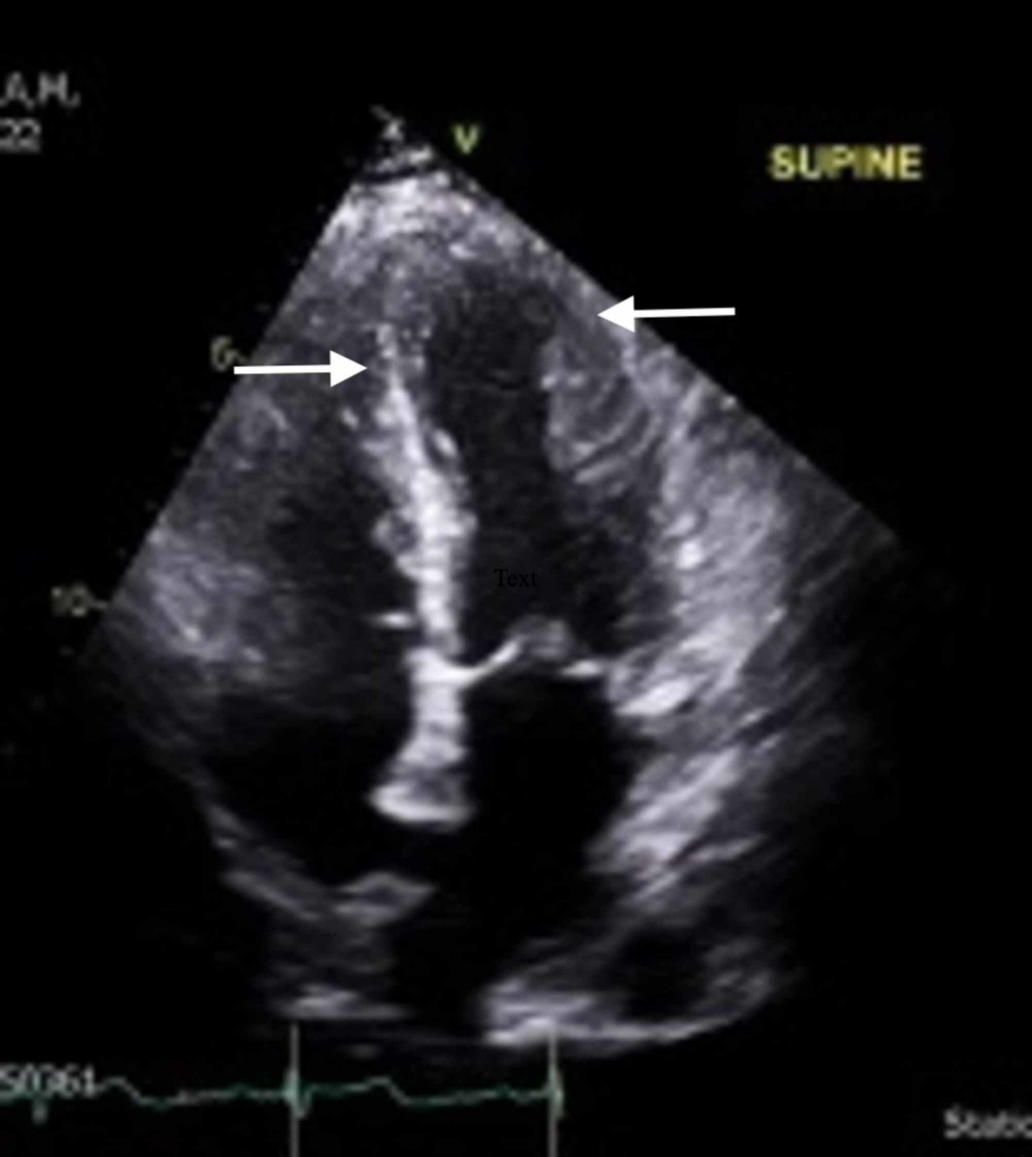
Transthoracic echocardiogram demonstrating mid to apical inferior and inferior lateral akinesis (white arrows)

**Figure 2:**
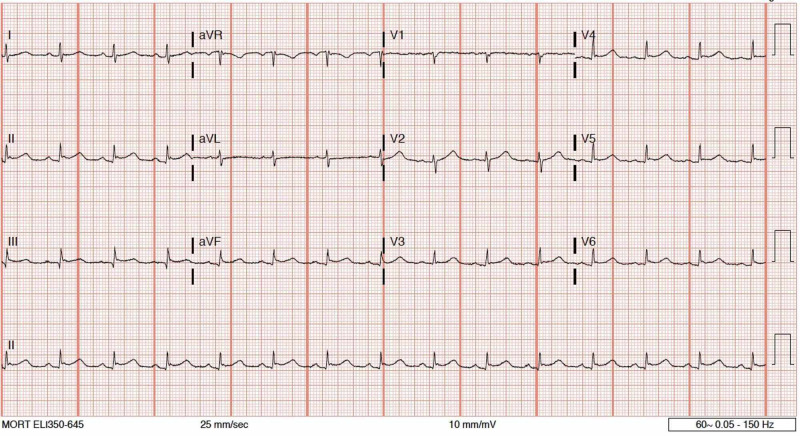
Electrocardiogram demonstrating diffuse ST-segment elevations without reciprocal depressions

During her ED course, the patient developed acute onset tachycardia up to 155 beats per minute and hypertension up to 185/116 mmHg, expanding the diagnostic investigation. Despite a non-ischemic-appearing electrocardiogram (ECG), the patient’s cardiac enzymes resulted in a troponin of 0.15 ng/mL. A demand mismatch was considered, but the patient’s history of hypercoagulopathy was concerning for a primary cardiac non-ST-segment elevation myocardial infarction (NSTEMI). Subsequently, the patient was started on a heparin drip and admitted to the intensive care unit. Continued inpatient workup with an echocardiogram revealed a reduced left ventricular systolic function with an ejection fraction of 41-51% and mid to apical inferior and inferolateral akinesis (Figure 1). Serial ECGs demonstrated interval development of inferior ST-segment elevations, and the patient underwent immediate cardiac angiography on hospital day 1 (Figure 2), which revealed a non-therapeutic catheterization and absent CAD. The patient was managed supportively and discharged after an uneventful hospital course, with scheduled outpatient follow-up with cardiology. The patient fully recovered to her pre-seizure baseline with the return of normal systolic cardiac function.

## Discussion

TCOM is a well-described mimicker of ACS that can be triggered by emotional or physical stress [1]. Previous examples of inciting factors include intense arguments, learning about serious diagnoses such as cancer, and physical exertion [3]. Though likely multifactorial, the predominant theory suggests that these inciters of TCOM cause an acute surge in catecholamines, with subsequent left ventricular dysfunction [3]. A less common etiology, however, is acute neurologic dysfunction; this includes intra-axial bleeding, ischemic strokes, migraines, and epilepsy [1,4]. Subarachnoid hemorrhages have the highest association, with TCOM developing in 15-25% of cases, compared to 1% of ischemic strokes [5,6,7]. 

Minimal data exist regarding the incidence of post-seizure TCOM, though a frequency of approximately 0.1% in post-seizure patients has been cited [8]. Although the pathophysiology of seizure-associated TCOM is less understood, a catecholaminergic surge during both the seizure itself and the postictal state is thought to be the underlying mechanism, with microvascular dysfunction and vasospasm likely contributing [9]. This hypothesis stems from the observation of prolonged, increased levels of noradrenaline in patients with prolonged seizures [10].

The presentations of TCOM can vary widely but typically mirror ACS; these include chest pain and anginal equivalents such as dyspnea, syncope, and near-syncope [11]. Specifically, a review of the International Takotsubo Registry (ITaK) demonstrated that chest pain (75.9%) and dyspnea (46.9%) are the most common symptoms [11]. Less frequently, patients will have symptoms consistent with acute heart failure exacerbation and hemodynamic instability [11]. Interestingly, data from the ITaK also suggest that patients with TCOM secondary to acute neurologic disease were significantly younger than patients with TCOM secondary to emotional stress and non-neurologic medical conditions or physical stressors, as in the case of our patient who developed TCOM at the age of 48 years [2]. In cases of seizure-induced TCOM, approximately 48% of symptoms occurred within five hours of seizure cessation, and the remaining 52% occurred in up to 288 hours after seizure resolution [12].

Diagnostic workup of patients with concern for TCOM includes ECG and cardiac enzymes. ECG often reveals ST-segment elevations, most commonly in the precordial leads, but can also reveal ST-segment depressions, deep and widespread T wave inversions, transient left bundle branch block, and arrhythmias [1,4]. Cardiac biomarkers, specifically troponins, can be significantly elevated and have been reported to be as high as 24 times the upper limit of normal [11]. Additional cardiac biomarkers including brain natriuretic peptide (BNP) can help to delineate TCOM versus true ACS, as BNP levels are disproportionately high in the former [13]. Continued workup as an inpatient with echocardiograms most commonly demonstrates apical ballooning of the left ventricle (LV) with associated hypokinesis, seen in approximately 82% of patients with TCOM [11]. Ultimately, the definitive diagnosis of TCOM is established with cardiac angiography and guidelines such as the Mayo Clinic criteria (Table 1) [10,14,15].

**Table 1:** Mayo Clinic criteria for TCOM [14] *There are rare exceptions in which the regional wall motion abnormality is limited to a single coronary artery perfusion territory; †It is possible to have a concurrent obstructive coronary atherosclerotic disease and TCOM TCOM: Takotsubo cardiomyopathy

Sr. no	Characteristics
1	Transient hypokinesis, akinesis, or dyskinesis of the left ventricular mid segments with or without apical involvement; the regional wall motion abnormalities extend beyond a single epicardial vascular distribution; a stressful trigger is often, but not always present*
2	Absence of obstructive coronary disease or angiographic evidence of acute plaque rupture^†^
3	New electrocardiographic abnormalities (either ST-segment elevation and/or T wave inversion) or modest elevation in cardiac troponin.
4	Absence of pheochromocytoma or myocarditis

We emphasize that in patients with ST-segment elevation meeting percutaneous coronary intervention (PCI) criteria, a clinical suspicion for TCOM should not delay coronary angiography or fibrinolysis [16,17]. Approximately 15% of patients with TCOM undergoing catheterization were found to have concomitant CAD [18]. Patients with elevated cardiac biomarkers not meeting PCI criteria should be treated similarly to primary cardiac NSTEMI, including anticoagulation, in conjunction with early cardiology consultation, especially in the setting of known ACS risk factors. Patients without elevated cardiac biomarkers are otherwise managed with supportive care. This includes initiation of beta-blockers or calcium channel blockers, and diuretics, and in those with severe hypokinesia or akinesia or LV thrombus, systemic anticoagulant therapy until the resolution of systolic dysfunction [10]. 

The prognosis of patients with TCOM who survive beyond the acute phase of the illness is favorable in terms of return of normal cardiac function, ranging from one to four weeks [19]. Echocardiographic abnormalities typically resolve within six weeks, and ECG abnormalities in 10 weeks [10]. Additionally, the rate of recurrence of TCOM also has been shown to be relatively low at 1.8% per patient-year [11]. Despite this favorable return of cardiac systolic function and low likelihood of recurrence, long-term follow-up of patients with TCOM demonstrated an all-cause mortality rate of 5.6% per patient-year and a rate of major adverse cardiac and cerebrovascular events of 9.9% per patient-year [11].

## Conclusions

Although TCOM is classically thought to stem from physical and emotional stressors, emergency providers should consider TCOM in patients with acute neurologic illness including seizures, and in those presenting with chest pain, heart failure, or hemodynamic instability. In these patients, emergency physicians should initiate a cardiac workup, including ECG, troponin, and BNP. New ST-segment elevations meeting PCI criteria should undergo cardiac catheterization to investigate ACS. Patients found to have TCOM should undergo cardiology follow-up until, at the minimum, the resolution of systolic dysfunction, and should be maintained on anticoagulation if severe LV systolic dysfunction or LV thrombus is present. Additionally, patients being discharged from the ED after a seizure should be given return precautions regarding the development of chest pain, anginal equivalents, or symptoms of acute heart failure given the possibility of delayed development of TCOM.
